# Case Report: Laryngeal web in a Yorkshire Terrier

**DOI:** 10.3389/fvets.2025.1595548

**Published:** 2025-07-21

**Authors:** Nicole Louie, Daniel Navarro Acosta, Russell Yeadon, Paweł M. Bęczkowski

**Affiliations:** ^1^Department of Veterinary Clinical Sciences, Jockey Club College of Veterinary Medicine and Life Sciences, City University of Hong Kong, Kowloon, Hong Kong SAR, China; ^2^Lumbry Park Veterinary Specialists, Alton, United Kingdom

**Keywords:** laryngeal web, larynx, congenital, ventriculocordectomy, dog, airways

## Abstract

The laryngeal web is a congenital or acquired, abnormal transverse membrane, most commonly between the free edges of the vocal cords. Ensuing glottic stenosis may be asymptomatic or, in some cases, can lead to vocal and respiratory signs, such as dysphonia, stridor, and dyspnoea. Here, we describe a rare case of a laryngeal web in a Yorkshire Terrier presented with atypical clinical signs, including dysphonia, flatulence, and halitosis. Following a reasonable exclusion of an initial concern of laryngeal neoplasia and given the diagnosis of a congenital laryngeal web, the owners elected not to pursue surgical treatment. The dog’s clinical signs remained unchanged with no development of new respiratory signs for over 4 years post-diagnosis. The findings presented herein highlight the importance of considering the laryngeal web as a rare but possible differential diagnosis for dysphonia and emphasise the need to establish specific individual selection criteria for surgical and medical treatments for dogs affected with this laryngeal anomaly.

## 1 Introduction

**Figure 1:**
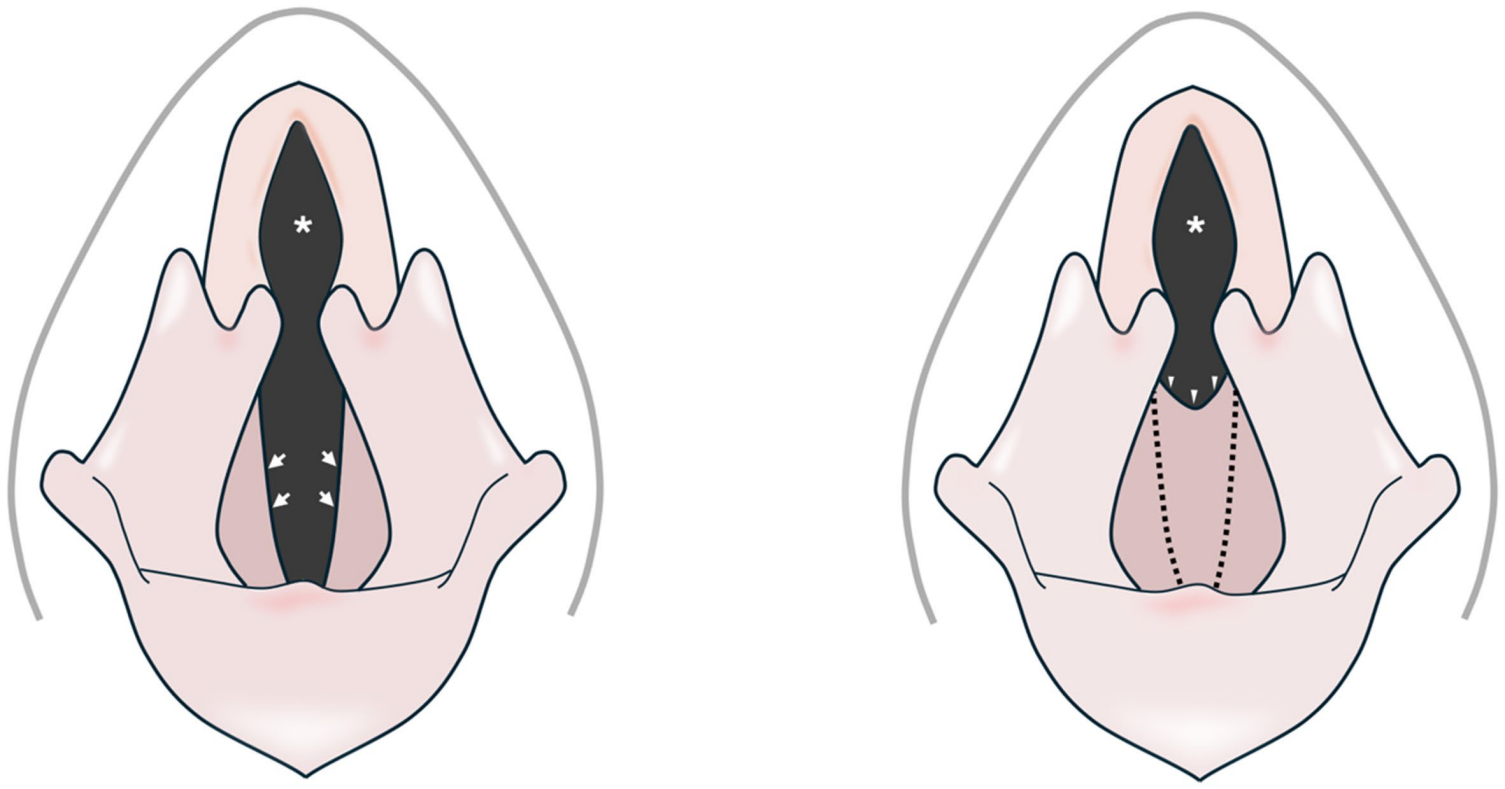
Schematic representation of a normal canine larynx (left) and one with a congenital web anomaly (right). The ventrolateral margins of the rima glottis of a normal canine larynx are bordered by the free edges of the vocal folds (arrows). A congenital laryngeal web defect (arrowheads) has replaced the normal free margins of the vocal folds (dotted lines), leading to a stenotic rima glottis (asterisks).

Laryngeal webs are predominantly discussed in human medical literature (1), but have also been described in animal species including dogs (2) and horses (3). Laryngeal webs in non-human species are typically characterised by an anomalous transverse membrane extending across the laryngeal lumen at or near the level of the vocal cords (1) (Figure 1). Supraglottic and subglottic webs have also been described in humans (4, 5). Laryngeal webs may be congenital or acquired in nature (1). Congenital webs are believed to result from the incomplete dissolution of fused embryonic vocal cords and are rarely described in the veterinary literature (3). Acquired laryngeal webs, commonly referred to as cicatrix formation (2), have been reported in dogs as a consequence of iatrogenic mucosal injury during ventriculocordectomy surgery (6). Ventriculocordectomy may be performed as a therapeutic procedure for conditions such as laryngeal paralysis or neoplasia (7). It may also be performed as a non-therapeutic procedure with the intent to diminish the volume and pitch of a dog’s bark (8, 9) - often referred to as “convenience” devocalisation. Due to welfare concerns, non-therapeutic ventriculocordectomy is legally prohibited in the European Union, the United Kingdom and numerous other jurisdictions (10–12). Acquired and congenital laryngeal webs can be asymptomatic or may present with vocal and respiratory signs, like dysphonia, stridor, and dyspnoea (2, 13). Treatment considerations for dogs primarily rely on the data from human medicine and historical reports of secondary, acquired laryngeal webs in dogs, encompassing both endoscopic and open surgical corrections (13–16). The present case report describes clinical signs, diagnosis and the management of suspected congenital laryngeal web in a dog. To the best of our knowledge, there are only two case reports of congenital laryngeal web in domestic animals, with no case documented since the 1980s (3, 17).

The case presented herein highlights the importance of considering this uncommon congenital anomaly in the list of differentials for dysphonia and upper airway obstruction in small animal patients.

## 2 Case description

### 2.1 History, clinical findings, and diagnostic investigations

An estimated 5-year-old, 3.95 kg, female intact Yorkshire Terrier who was rescued couple of months ago, was presented to a primary care veterinarian for routine neutering and dental treatment, during which an obstruction at the larynx was identified, preventing scheduled intubation. The patient received routine ovariohysterectomy and dental treatment including multiple dental extractions utilising an undersized endotracheal tube (ETT) (2.5 mm) without complications. Clinically, the dog could not bark, producing an atypical squeaky breathing noise. There was significant flatulence and halitosis, but no stertor, stridor, exercise intolerance, or other significant abnormalities were detected on the physical examination. The flatulence was reported to occur specifically when attempting to bark. The dog was subsequently referred for further investigation into suspected laryngeal neoplasia as the cause of dysphonia.

**Figure 2:**
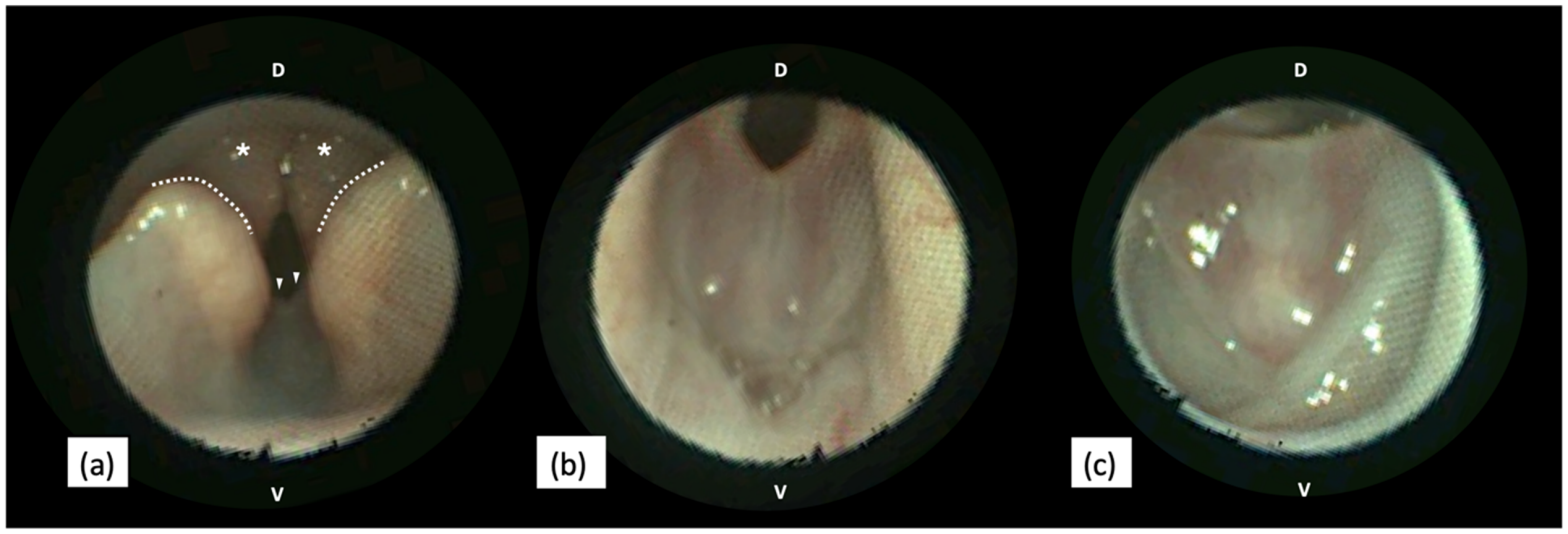
Endoscopic views of a canine larynx presenting a congenital web anomaly. **(a)** In the rostral view, the web defect (arrowheads) is visible caudal to the cuneiform processes (dotted line), aligned with the corniculate processes (asterisks) of the arytenoid cartilages. **(b,c)** Close-up views of the web anomaly during exhalation **(b)** and inhalation **(c)**. During inhalation, the free edge of the membranous web is stretched as the larynx abducts **(c)**. D, Dorsal. V, Ventral. Images were obtained with KARL STORZ SILVER SCOPE® Veterinary Video Endoscope (KARL STORZ SE & Co. KG, Tuttlingen, Germany).

Complete blood count and serum biochemistry analysis were unremarkable. A video laryngoscopy was performed upon induction of general anaesthesia, revealing a pink, smooth, regular, membranous web in the ventral glottis extending between the vocal cords and resulting in a stenotic rima glottis (Figure 2). Intubation was achieved without difficulty with a 4 mm ETT; however, the membrane could not be moved, necessitating a more dorsal tube placement than usual. Subsequent endoscopic examination of the trachea was unremarkable.

The following differential diagnoses for glottic stenosis were considered: exuberant granulation or fibrotic tissue secondary to traumatic or iatrogenic mucosal injury, obstructive laryngitis, everted laryngeal saccules, and neoplasia. Based on the clinical history and endoscopic findings, glottic stenosis due to a congenital laryngeal web was the most likely diagnosis. This finding was communicated to the dog’s caregiver during the endoscopy; the owner declined further investigations.

### 2.2 Management and outcomes

Since the presenting clinical signs for the dog were not considered to be affecting the quality of life, and the initial concern regarding a differential diagnosis of laryngeal neoplasia was considered to have been reasonably excluded, the owners elected not to pursue further specific diagnostics and treatment. Financial constraints and concerns regarding possible surgical complications were additional considerations when electing against surgical management. The owner was contacted by e-mail just over 4 years after the initial presentation and confirmed that the dog’s clinical signs remained unchanged, with non-progressive dysphonia and no development of new respiratory signs such as dyspnoea or coughing. The flatulence when attempting to bark continued, as previously reported. It was not possible to reliably establish whether significant halitosis was an ongoing feature.

## 3 Discussion

Laryngeal webs can be congenital or more commonly acquired as a consequence of traumatic or iatrogenic mucosal injuries (6, 18, 19). Most dogs with reported laryngeal web had a history of laryngeal procedures where mucosal defects were left to heal by secondary intention without primary closure (13). Laryngeal cicatrix formation is a well-documented post-operative complication of ventriculocordectomy *via* oral approach or ventral laryngotomy, unilateral arytenoidectomy, bilateral laryngeal sacculectomy, and castellated laryngofissure with vocal fold resection (2, 6, 13, 14). Although it cannot be confidently ruled out that the recent stray dog presented in our case report had not undergone any laryngeal procedure in the past, it is important to emphasise that a ventriculocordectomy or vocal cordectomy for canine devocalisation has been prohibited in the UK since 2006 (10), which predates the dog’s estimated birth by at least 9 years. Additionally, there were no clinical findings or alterations in laryngeal anatomy on laryngoscopy that might indicate a history of laryngeal paralysis or collapse that might have necessitated previous therapeutic ventriculocordectomy. This leads to a valid suspicion that the case presented in this report was affected by a congenital laryngeal web.

Embryological morphogenesis and development of the larynx share many similarities amongst mammalian species (20). Laryngeal web anomalies are thought to arise from an unsuccessful dissolution of fused embryonic vocal cords and, therefore, incomplete recanalization of the larynx (3). In humans, congenital laryngeal webs are rare, with an incidence of 1 in 10,000 births, representing 5% of all laryngeal defects (18, 21). Congenital webs in humans can be membranous or associated with aberrant soft tissues; they typically develop at the glottic level at the anterior commissure, analogous to the ventral glottis in animals, but may extend into the subglottic or supraglottic region (5, 18, 19). Descriptions of laryngeal web morphology in dogs are limited to acquired cases, identified as protruding cicatrix, granulation, or fibrous scar tissues that often asymmetrically span the rima glottidis (2, 6, 13, 14, 22, 23). In our case, the morphological characteristic of the web was distinct; it was a symmetrical, thin, and membranous web localised to the ventral glottis bridging the vocal folds, resembling the gross appearance of a membranous laryngeal web in humans.

In dogs, there is a single case report documenting a congenital laryngeal web defect in a 5-month-old mixed-breed presented with dyspnoea and toneless barking (17). The dog exhibited simultaneous bilateral arytenoid hypoplasia, abnormal intrinsic laryngeal muscles, and a dome-shaped cricoid cartilage, unlike our case, which presented solely with a laryngeal web. Concurrent anomalies were also found in a 10-day-old Quarter Horse filly, including dorsal displacement of the soft palate, aberrant laryngeal ventricles, and epiglottic hypoplasia (3). Examples of concomitant lesions in humans with congenital laryngeal web included congenital heart disease, subglottic stenosis independent of the web, and velocardiofacial gene deletion syndrome (18). Whilst, based on a thorough clinical examination, concurrent congenital anomalies in our case were thought to be unlikely, the lack of advanced imaging and investigations other than endoscopy left this rather improbable prospect unexamined. Still, the likelihood of concomitant abnormalities or disorders should be considered as a possibility in animals with congenital laryngeal webs. Laryngeal paralysis and collapse are often present in conjunction with acquired laryngeal web, requiring a thorough dynamic laryngoscopy to rule out synchronous functional airway obstructions (13, 14). In the dog reported, we did not identify any other airway abnormalities or other clinical anomalies, including on full upper airway endoscopy.

Clinically, dogs with a laryngeal web often present with respiratory and vocal signs. Dyspnoea, inspiratory stridor, and exercise intolerance are the most commonly reported signs in acquired cases; however, they could also display a toneless bark and cough after drinking (6, 13, 14, 17). The case presented in this report was referred due to suspicion of laryngeal neoplasia as a cause of dysphonia, flatulence and halitosis. Subsequent diagnosis of the laryngeal web highlights that this rare congenital condition must be considered as a possible differential diagnosis for dysphonia. Although flatulence has not been reported as a clinical sign in animals or humans with laryngeal webs, it may be explained by aerophagia from the dog’s persistent effort to bark. It is unclear whether halitosis could be attributed to the laryngeal web and aerophagia or simply caused by a prior severe periodontal disease.

Endotracheal intubation can be complicated by the presence of a laryngeal web, as demonstrated in our case. There are several alternatives to secure the airways in dogs with laryngeal webs, including an anaesthetic mask, the peri-cuff sealing technique caudal to the laryngeal web, or even tracheostomy (6). Whilst no recommendations for intubation of dogs with laryngeal web can be made from our case, we achieved satisfactory intubation by employing a smaller-than-usual ETT without intra or post-procedural complications.

Laryngoscopy appears to be a sufficient method for identifying the laryngeal web; cytological or histopathological evaluation is typically not required to diagnose this structural anomaly (2, 6, 13). In our case, given the small size of the dog and the original suspicion of laryngeal neoplasia, we utilised the video endoscopy to thoroughly examine the airways. Its ability to magnify and directly visually assess the laryngeal structures under high resolution facilitates real-time evaluation, which is also critical for accurately ruling out dynamic upper airway obstructions (24). In this case, samples were not obtained for cytology or histopathology as the videoscopic appearance was highly suggestive of a congenital laryngeal web and the consideration of potential complications of laryngeal sampling. Biopsy, lymph node examination, and at least thoracic radiography are necessary when the web morphology resembles neoplastic or inflammatory lesions, particularly for acquired webs, as differentiation based solely on gross appearance may not be possible (23).

In humans, Cohen’s classification (Type I to IV), which quantifies web severity based on the percentage blockage of the glottis, is most commonly applied (25). Nonetheless, the extrapolation of this classification may prove impractical in veterinary medicine owing to the difference in canine laryngeal anatomy. The case review of human paediatric patients with congenital and acquired laryngeal web indicates that stratifying patients by the severity of the laryngeal web, rather than aetiology, is a more significant predictor of clinical complexity (18). Over 30% of human patients with Type I and II (<35% and 35–50% of glottic blockage) can be managed conservatively through long-term observations (18). Those findings suggest evaluating the severity of vocal and respiratory signs individually when considering treatments for veterinary patients. In our case, the dog’s respiratory function remains unaffected by the web defect, with a residual stable non-progressive dysphonia. Clinical stability 4 years after the diagnosis indicates that conservative management, which in our case involved watchful surveillance without any surgical or medical intervention, may be a viable option for most dogs with mild clinical signs associated with laryngeal web formation. It remains unknown, however, whether this approach also applies to all dogs, particularly those with brachycephalic obstructive airway syndrome (BOAS), where early surgical intervention to address narrowed airways is recommended to improve outcomes (26). Surgical management of laryngeal webs in dogs has been reported previously (13–15), and whilst it is commonly associated with a favourable prognosis, post-surgical web recurrence is possible (13, 18). In some cases, the presence of coinciding airway obstructions, such as concurrent laryngeal paralysis, is associated with long-term complications, including refractory exercise intolerance and stridor (2, 13, 18).

In conclusion, this case report indicates that congenital laryngeal web should be considered a possible differential diagnosis in dogs with dysphonia alongside more common differential diagnoses such as laryngeal inflammation or neoplasia. The dog presented herein was managed conservatively, with clinical signs of dysphonia remaining unchanged, with no development of new respiratory signs for over 4 years since the diagnosis. Whilst laryngeal webs can be managed medically or surgically as informed by the clinical severity, specific selection criteria for the individual dogs remain to be established.

## Data Availability

The original contributions presented in this case report are included in the article. Any further inquiries can be directed to the corresponding author.
